# HPValidate—human papillomavirus testing with DNA and mRNA assays on self-collected samples in cervical screening: comparison of test characteristics on three self-sampling devices

**DOI:** 10.1038/s41416-025-03102-5

**Published:** 2025-07-08

**Authors:** Christopher S. Mathews, Alexandra Sargent, Kate Cuschieri, Matejka Rebolj, Adam R. Brentnall, Anne Mackie, Charlotte Mills, Carolina Martinelli, Ann-Marie Wright, Katherine Hunt, Andrew Bird, Hasit Patel, David Smith, Trudy Johnson, Kay Ellis, Mark Hunt, Karin Denton

**Affiliations:** 1 Centre for Cancer Screening, Prevention and Early Detection, Wolfson Institute of Population Health, Queen Mary University of London, London, UK; 2 Cytology Department, Manchester University NHS Foundation Trust, Manchester, UK; 3 Scottish HPV Reference Laboratory, Royal Infirmary of Edinburgh, NHS Lothian Scotland, Edinburgh, UK & Centre for Reproductive Health, University of Edinburgh, Edinburgh, UK; 4 Centre for Evaluation and Methods, Wolfson Institute of Population Health, Queen Mary University of London, London, UK; 5 Primary Care and Prevention, Department of Health and Social Care, London, UK; 6 Severn Pathology, Southmead Hospital, North Bristol NHS Trust, Bristol, UK; 7 Health Service Laboratories LLP, London, UK; 8 NEY Cervical Screening, The NHS Pathology Centre, Queen Elizabeth Hospital, Gateshead Health NHS Foundational Trust, Gateshead, UK; 9 Cytopathology Department, Norfolk and Norwich University Hospitals NHS Foundation Trust, Norwich, UK; 10 Present Address: Birmingham and Solihull Integrated Care Board, NHS Birmingham and Solihull, Birmingham, UK

**Keywords:** Cancer screening, Cancer screening

## Abstract

**Background:**

Relative test accuracy of human papillomavirus (HPV) testing on self vs. clinician-collected samples may depend on the specific combination of a self-sampling device and HPV assay.

**Methods:**

Five self-sampling workflows were studied within the routine English cervical screening programme; the cobas HPV DNA and APTIMA HPV mRNA assays with the Evalyn brush, Self Vaginal FLOQSwabs (FLOQSwabs) and the Multitest kit. To study test sensitivity, women were recruited at routine colposcopy appointments; to study test specificity, women were recruited at routine screening appointments.

**Results:**

The estimated conditional relative sensitivity for high-grade cervical intraepithelial neoplasia (CIN2+) was 0.90 (90% CI: 0.84–0.94) for the Evalyn + cobas workflow, 0.94 (0.90–0.97) for FLOQSwabs + cobas, 0.77 (0.69–0.83) for Evalyn + APTIMA, 0.92 (0.85–0.96) for FLOQSwabs+APTIMA and 0.92 (0.86–0.96) for Multitest+APTIMA. The estimates of the relative specificity were 0.96 (0.95-0.98), 0.91 (0.90-0.93), 0.99 (0.97–1.01), 0.89 (0.87–0.92) and 0.87 (0.85–0.89), respectively. The specificity estimates were sensitive to the inclusion of certain subgroups of women. HPV detection rates were higher for all self-sample than clinician-sample workflows.

**Conclusions:**

The relative test sensitivity of four self-sampling workflows including both DNA and mRNA HPV assays was relatively close to that associated with clinician-collected samples.

## Background

Human papillomavirus (HPV) testing on self-collected samples has the potential to increase screening uptake and offer women more choice of screening options [1–3], and some countries have introduced HPV self-collection in their cervical screening service [4]. In this context, HPV assays that were originally optimised and validated for clinician-collected samples are being repurposed for the testing of self-collected samples. However, few have a regulatory approval to be used in screening with self-samples [5]. There is also a relatively small number of self-sampling devices that have a generic claim/CE mark for use in the home environment for downstream HPV testing [6].

A key condition for self-collection to have a role in a screening service is test accuracy for the detection of high-grade cervical intraepithelial neoplasia (CIN2+ or CIN3+). Several studies have been undertaken to help understand this, some of which recruited women attending for primary screening [7, 8]. Overall, previous work has identified that accuracy may depend on the assay, the self-sampling device [9] and sample processing protocols [10]. Hence, further studies are required for screening services to gain a more complete overview of the available options, particularly those where the end-to-end process, which includes the pre-analytical phase, is established, transparent and consistently applied.

In 2018, the UK National Screening Committee recommended that self-sampling required further study in well organised pilots and research projects [11]. NHS England is now considering an introduction of self-sampling as an offer for all women. To inform the choice of self-sampling device and test for such an implementation, the screening programme commissioned the ‘HPValidate’ study reported here. This was a pragmatic study designed to compare the relative sensitivity and specificity of self-collected vs. clinician-collected samples for the detection of CIN2+ as well as other operational characteristics for five device-assay combinations (‘workflows’; Table 1) [12–14]. The study included the two HPV assays that are used currently for the routine testing of clinician-collected cervical samples in England, cobas (Roche, Rotkreuz, Switzerland; which detects HPV DNA) and APTIMA (Hologic, Marlborough, MA; which detects HPV mRNA) [15], and three self-sampling devices: Self Vaginal FLOQSwabs (FLOQSwabs; Copan Italia, Brescia, Italy) and Evalyn (Rovers Medical Devices, Oss, The Netherlands), both transported dry, in addition to one wet device, the Aptima Multitest Swab (Hologic, Marlborough, MA).

**Table 1:** The five self-sampling workflows studied in HPValidate and description of the recruited women, by self-sampling workflow.

	Self-sampling workflow					
	Evalyn + cobas	FLOQSwabs + cobas	Evalyn + APTIMA	FLOQSwabs + APTIMA	Multitest + APTIMA	P
**Colposcopy population**						
Inclusion criteria	Referred to colposcopy for further investigation after a positive or inadequate HPV test result at screening or early recall at most 8 weeks (≤56 days) before the colposcopy appointment					
Exclusion criteria	Unable to provide consent; referred after primary care have unsuccessfully been able to obtain a sample; reason for referral was a negative HPV test result; attending for test of cure or continued management					
Laboratory (number of recruitment sites)	Gateshead NHS Foundation Trust; Norfolk and Norwich University Hospitals NHS Foundation Trust (4)	Manchester University NHS Foundation Trust (2)	North Bristol NHS Trust (2)	Health Services Laboratories, London (1)	Health Services Laboratories, London; North Bristol NHS Trust (2)	
N recruited	471	351	391	132	212	
N eligible^a^	102 (100%)	120 (100%)	86 (100%)	66 (100%)	78 (100%)	
Age range, median (IQR)^b^	24–59, 34.5 (29.3–40.0)	24–67, 35.0 (30.0–40.0)	24–58, 33.5 (28.0–40.5)	24–60, 32.0 (28.0–36.0)	24–63, 31.5 (27.0–39.8)	0.12
Directly referred (%)^b^	89 (87.3%)	70 (58.3%)	74 (86.0%)	66 (100%)	74 (94.9%)	<0.001
Referral cytology negative^b^	7 (6.9%)	8 (6.7%)	7 (8.1%)	0 (0%)	4 (5.1%)	
Referral cytology borderline or low-grade abnormal^b^	18 (17.6%)	9 (7.5%)	40 (46.5%)	0 (0%)	19 (24.4%)	
Referral cytology high-grade abnormal^b^	77 (75.5%)	102 (85.0%)	39 (45.3%)	66 (100%)	55 (70.5%)	
Referral cytology inadequate^b^	0 (0%)	1 (0.8%)	0 (0%)	0 (0%)	0 (0%)	
Abnormal referral cytology (%)^b^	95 (93.1%)	111 (92.5%)	79 (91.9%)	66 (100%)	74 (94.9%)	<0.001
**Primary screening population**						
Inclusion criteria	Due or overdue for the routine cervical screening test at 24–64 years and will have already received their invitation letter					
Exclusion criteria	Unable to provide consent; presenting before their next test due date, including clinical referrals; on early recall due to a previous HPV positive result					
Laboratory (number of recruitment sites)	Gateshead NHS Foundation Trust; Norfolk and Norwich University Hospitals NHS Foundation Trust (8)	Manchester University NHS Foundation Trust (5)	North Bristol NHS Trust (5)	Health Services Laboratories, London (11)	Health Services Laboratories, London (9)	
N recruited	945	1010	970	895	1035	
N eligible^c^	940 (100%)	994 (100%)	909 (100%)	878 (100%)	1006 (100%)	
Age range, median (IQR)^b^	24–64, 41.0 (33.0–50.0)	24–64, 39.0 (32.0–48.0)	24–64, 41.0 (33.0–49.0)	24–64, 35.0 (29.0–45.0)	24–64, 37.0 (30.0–45.0)	<0.001
No abnormality in screening history (%)^b^	929 (98.8%)	974 (98.0%)	880 (96.8%)	795 (90.5%)	905 (90.0%)	<0.001

*HPV* human papillomavirus, *IQR* interquartile range.

^a^Reasons for exclusion from the analyses (for all five workflows combined): 2 (0.1%) with incomplete or withdrawn consent; 19 (1.2%) because of incomplete HPV test results on the self-collected samples; 2 (0.1%) because of incomplete clinician-collected HPV test results; 24 (1.5%) with the referral LBC sample taken >56 days prior or at an unknown date; 19 (1.2%) presenting for an unrelated reason; 4 (0.3%) because of other unresolved errors; and 1035 (66.5%) because their diagnosis was <CIN2.

^b^Among those included in the analyses.

^c^Reasons for exclusion from the analyses (for all five workflows combined): 10 (0.2%) with incomplete or withdrawn consent; 6 (0.1%) because they were outside of the target age range for screening; 66 (1.4%) because of incomplete HPV test results on the self-collected samples; 4 (0.1%) because of incomplete clinician-collected HPV test results; and 42 (0.9%) because they had a CIN2+ diagnosis after direct referral.

Here, we estimate the relative sensitivity and specificity for the detection of CIN2+ of the five self-sampling workflows included in the HPValidate study, in referral and primary screening settings, respectively.

## Methods

### Cervical screening in England; parameters and protocols

In England, women aged 24.5–49 years are invited for cervical screening every 3 years; between 50 and 64 years this changes to invites every 5 years. Since late 2019, all primary screening has been based on HPV testing using ThinPrep PreservCyt (Hologic, Marlborough, MA) liquid-based cytology (LBC) samples collected by a clinician [16]. The programme has published a list of HPV assays which have been accepted for use according to national protocols [17]. Two HPV tests from this list are currently in use; the cobas HPV test on the 6800 or 8800 platform and the APTIMA HPV Assay on the APTIMA Panther platform [15]. Collection and processing of the samples follow national guidelines and manufacturers’ instruction for use [17–19].

Reflex cytology is performed on all samples that return a positive test result for at least one of the 14 high-risk HPV genotypes. Women with at least borderline abnormalities are directly referred to colposcopy. Those with negative cytology are referred to early recall in 12 months, and to another early recall in 12 months if persistently HPV-positive with negative cytology. A referral to colposcopy is made at 12 months after screening when the early recall test is HPV positive and cytology has progressed to abnormal; or at 24 months in case of a third positive HPV test regardless of the concurrent cytology (note that HPV genotyping does not influence pathways for clinical management) [20]. The national guidance requires that women with high-grade abnormal cytology are seen in colposcopy within 2 weeks and those with less severely abnormal cytology within 6 weeks [21]. Women with a negative HPV test at primary screening or early recall are returned to age-appropriate 3- or 5-year routine recall.

### Study design

To study conditional relative test sensitivity, women were recruited when they attended their routine colposcopy appointments following a positive clinician-collected HPV test from primary screening or early recall that had been taken, at most, 8 weeks before (Table 1). To study conditional relative test specificity, women were recruited at primary care when they presented for screening following an invitation. In both settings, women were recruited consecutively, although the numbers and reasons for refusal to participate could not be registered due to administrative capacity. The recruitment took place between July 2021 and September 2023, i.e. after the initial disruption to screening services due to the first COVID-19 pandemic lockdown [22]. After informed consent, women were asked to provide a self-collected sample using device-specific instructions [23–25]. Self-collected samples were handed to the clinician and were transported, without any processing at the collection site, to the participating laboratories alongside other routine samples. HPV test results from self-collected samples were not shared with women or their clinicians. All clinician-led investigations were completed after self-collection. Clinician collection and further clinical management were unaffected by the study and followed the relevant national and/or local guidance.

Information on CIN2+ diagnoses was retrieved from colposcopy records available routinely to screening laboratories. In women recruited in primary care, this only included women who were referred directly after an HPV-positive primary screening test and abnormal cytology triage. Direct referral cases represent around two-thirds of all CIN2+ diagnosed after a positive HPV test [26], but information on CIN2+ from early recall would delay the completion of the study by another 2 years. We treated women who were HPV-negative on the clinician sample as having no CIN2+ in the analysis.

**Fig. 1:**
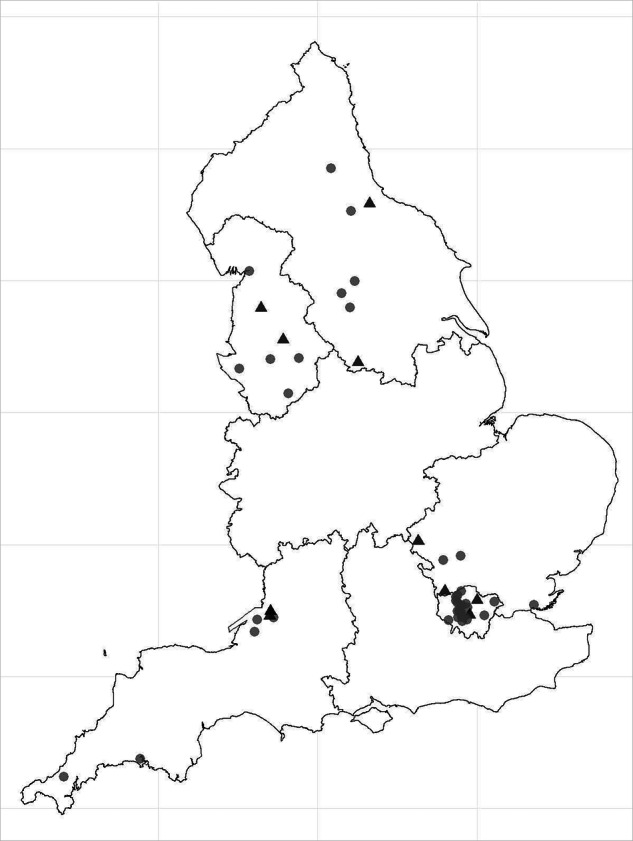
Recruitment site locations for the HPValidate study. Triangles: colposcopy units. Circles: primary care practices.

To operationalise the five self-sampling workflows, the laboratories used the HPV assay in use for routine screening in their context and were assigned one self-sampling device. The paired self- and clinician-collected samples were tested with the same HPV assay. Each workflow was performed in one laboratory, but two workflows had a second laboratory assigned to help with recruitment targets (Table 1). Women were recruited from primary care sites and colposcopy clinics in these laboratories’ routine service catchment areas (Fig. 1).

### Laboratory processes and sample handling

After samples collected with the Evalyn or FLOQSwabs devices were removed from their casings in the laboratory, they were resuspended in 5 ml ThinPrep PreservCyt and vortexed for 2 × 15 s. Immediately thereafter, the resuspended FLOQSwabs was introduced to the cobas 6800/8800 or 1 ml was transferred into a labelled APTIMA Specimen Transfer tube pre-filled with 2.9 ml STM for testing on the APTIMA Panther. For the resuspended Evalyn brushes 1 ml was transferred into a second labelled tube for testing with the cobas 6800/8800 or to a labelled APTIMA Specimen Transfer tube for testing on the APTIMA Panther. Prior to introducing samples to the Panther instrument, all samples (collected either with Evalyn, FLOQSwabs, or Multitest devices) were pre-heated at 90 °C for 75 min using a hot block (during this process, all tubes remained capped) and were thereafter allowed to cool. After pre-analytic processing, HPV testing was carried out as per manufacturers’ instructions for use information for ThinPrep PreservCyt cervical taken samples. Samples returning an invalid test result on the first run were retested once; for those samples, the result obtained from the second run was reported.

### Analysis

Conditional relative sensitivity of each self-sampling workflow was defined as the proportion of CIN2+ cases detected through a positive clinician-collected sample that also returned a positive HPV test result on a self-collected sample. As we discussed previously [27], this approach substantially reduces the required study size without necessitating a change in clinical management. However, it leads to conservative sensitivity estimates and is therefore useful for accepting a good screening test. Two-sided 90% confidence intervals (CI) were calculated using Wilson’s method for a binomial parameter. This was done because primary interest was in the lower tail (i.e. a 1-sided *α* = 0.05). These calculations were repeated for the following subgroups of women: those with CIN3+, with high-grade abnormal referral cytology and after adding CIN2+ cases excluded from the analysis of test specificity.

To estimate the conditional relative specificity in women without CIN2+, the number in the primary screening setting with a negative self-sampling test result was compared with the number having a negative test on the paired clinician-collected sample. Two-sided 90% CI were calculated as given by Hayen et al. [28], using the same reasoning as above. These calculations were repeated after exclusion of women with inadequate self-sampling tests, and of women aged <30 years or those with recent abnormalities.

Differences in the distribution of women’s characteristics between the workflows were evaluated using the Kruskal-Wallis test for continuous variables (age) and chi-square tests for categorical variables (proportions with a direct referral, with abnormal referral cytology, or with recent abnormalities), with p-values for the latter obtained through three million simulated replications. The trend in the proportion of positive tests in women with CIN2+ by age and cytological abnormality was assessed with a one-sided Armitage-Cochran test for trend. Because the prevalence of HPV infections decreases as women age, the relative specificity is expected to increase with age, unrelated to HPV testing technology. In the primary screening populations, therefore, we focused on age-specific changes in the concordance of HPV test results between the two sample types (calculated as: sum of concordant tests/total number of women) using the Wilcoxon rank sum test [29].

Sample size targets were pre-planned and are justified in Supplementary Information. The target study size to estimate test sensitivity was ≥60 women with CIN2+ per workflow. Based on previous data from the English screening programme [26, 30], it was considered that this number should be achieved after at most 350 consecutive colposcopy referrals. To study test specificity, the study sought to recruit 1000 consecutive women per workflow. Neither conditional relative sensitivity nor specificity were (a priori) powered to test for heterogeneity in any subgroup analysis.

All analyses were undertaken with R version 4.2.2.

## Results

### Women recruited into the study

Table 1 shows the number recruited and eligible for analysis by workflow, including reasons for exclusion. Among women recruited at colposcopy clinics, there were significant differences between the five workflows in the reason for the referral and the proportions with cytological abnormalities at the referral. In primary screening, the five workflows differed in women’s ages and screening histories.

### Relative sensitivity

Positivity of self-collected HPV test samples in women with CIN2+ is reported by workflow in Table 2; estimates of conditional relative sensitivity are given in Table 3. In the primary analysis including all women with CIN2+ from the colposcopy referral population, four workflows: Evalyn + cobas, FLOQSwabs + cobas, FLOQSwabs + APTIMA and Multitest + APTIMA achieved a relative sensitivity ranging between 0.90 (Evalyn+cobas) and 0.94 (FLOQSwabs+cobas), with the lower bounds of the 90% CI ranging between 0.84 and 0.90, respectively. The Evalyn+APTIMA workflow had the lowest relative sensitivity (0.77, 90% CI: 0.69–0.83). These estimates remained similar in subgroup analyses. The data could not confirm a trend in the relative test sensitivity by age or cytology grade at referral, though the numbers were small (Table 4).

**Table 2:** HPV test results on the linked self- vs. clinician-collected samples, by self-sampling workflow.

	Colposcopy population (women with CIN2+)	Primary screening population (women without CIN2+)			
	LBC positive	LBC negative	LBC positive	LBC invalid	Total
Evalyn + cobas					
SS negative	10 (9.8%)	785 (83.5%)	11 (1.2%)	0 (0%)	796 (84.7%)
SS positive	92 (90.2%)	35 (3.7%)	100 (10.6%)	0 (0%)	135 (14.4%)
SS invalid	0 (0%)	8 (0.9%)	1 (0.1%)	0 (0%)	9 (1.0%)
Total	102 (100%)	828 (88.1%)	112 (11.9%)	0 (0%)	940 (100%)
FLOQSwabs + cobas					
SS negative	3 (2.5%)	822 (82.7%)	11 (1.1%)	0 (0%)	833 (83.8%)
SS positive	113 (94.2%)	35 (3.5%)	70 (7.0%)	0 (0%)	105 (10.6%)
SS invalid	4 (3.3%)	55 (5.5%)	1 (0.1%)	0 (0%)	56 (5.6%)
Total	120 (100%)	912 (91.8%)	82 (8.2%)	0 (0%)	994 (100%)
Evalyn + APTIMA					
SS negative	20 (23.3%)	759 (83.5%)	37 (4.1%)	5 (0.6%)	801 (88.1%)
SS positive	66 (76.7%)	51 (5.6%)	56 (6.2%)	0 (0%)	107 (11.8%)
SS invalid	0 (0%)	1 (0.1%)	0 (0%)	0 (0%)	1 (0.1%)
Total	86 (100%)	811 (89.2%)	93 (10.2%)	5 (0.6%)	909 (100%)
FLOQSwabs + APTIMA					
SS negative	5 (7.6%)	665 (75.7%)	22 (2.5%)	0 (0%)	687 (78.2%)
SS positive	61 (92.4%)	105 (12.0%)	86 (9.8%)	0 (0%)	191 (21.8%)
SS invalid	0 (0%)	0 (0%)	0 (0%)	0 (0%)	0 (0%)
Total	66 (100%)	770 (87.7%)	108 (12.3%)	0 (0%)	878 (100%)
Multitest + APTIMA					
SS negative	5 (6.4%)	771 (76.6%)	12 (1.2%)	1 (0.1%)	784 (77.9%)
SS positive	72 (92.3%)	132 (13.1%)	89 (8.8%)	0 (0%)	221 (22.0%)
SS invalid	1 (1.3%)	1 (0.1%)	0 (0%)	0 (0%)	1 (0.1%)
Total	78 (100%)	904 (89.9%)	101 (10.0%)	1 (0.1%)	1006 (100%)

*CIN* cervical intraepithelial neoplasia, *LBC* liquid-based cytology clinician-collected sample, *SS* self-collected sample.

**Table 3:** Estimates of the conditional relative sensitivity and specificity, by self-sampling workflow.

	Evalyn + cobas		FLOQSwabs + cobas		Evalyn + APTIMA		FLOQSwabs + APTIMA		Multitest + APTIMA	
	n/N^a^	Estimate (90% CI)	n/N^a^	Estimate (90% CI)	n/N^a^	Estimate (90% CI)	n/N^a^	Estimate (90% CI)	n/N^a^	Estimate (90% CI)
Relative sensitivity										
All, CIN2+^b^	92/102	0.902 (0.843–0.940)	113/120	0.942 (0.896–0.968)	66/86	0.767 (0.685–0.834)	61/66	0.924 (0.852–0.963)	72/78	0.923 (0.858–0.960)
All, CIN3+	41/45	0.911 (0.816–0.959)	75/77	0.974 (0.925–0.991)	36/47	0.766 (0.652–0.851)	17/19	0.895 (0.727–0.965)	31/31	1 (0.920–1.000)
High–grade cyt, CIN2+	71/77	0.922 (0.856–0.959)	96/102	0.941 (0.890–0.969)	27/39	0.692 (0.562–0.798)	61/66	0.924 (0.852–0.963)	50/55	0.909 (0.825–0.955)
All + primary, CIN2+	95/106	0.896 (0.837–0.935)	121/128	0.945 (0.902–0.970)	74/96	0.771 (0.693–0.833)	68/73	0.932 (0.866–0.966)	85/91	0.934 (0.878–0.966)
Relative specificity										
All, CIN2+^b^	796/828	0.961 (0.947–0.976)	833/912	0.913 (0.896–0.931)	801/811	0.988 (0.968–1.007)	687/770	0.892 (0.870–0.915)	784/904	0.867 (0.847–0.888)
Adequate HPV SS tests, CIN2+	796/820	0.971 (0.957–0.984)	833/857	0.972 (0.959–0.985)	801/810	0.989 (0.970–1.008)	687/770	0.892 (0.870–0.915)	784/903	0.868 (0.848–0.889)
≥30 years, no recent abn, CIN2+	680/703	0.967 (0.953–0.982)	691/752	0.920 (0.900–0.938)	695/702	0.990 (0.971–1.010)	500/530	0.943 (0.921–0.966)	598/673	0.889 (0.866–0.911)

*abn* abnormalities, *CI* confidence interval, *CIN* cervical intraepithelial neoplasia, *cyt* cytology, *HPV* human papillomavirus, *primary* including CIN2+ cases diagnosed after direct referral in women recruited at primary care, *SS* self-collected samples.

^a^Relative sensitivity: n = number of women with a positive HPV test result on the self-collected sample, N = number of women with a positive HPV test result on the clinician-collected referral sample, among those with CIN2+. Relative specificity: n = number of women with a negative HPV test result on the self-collected sample, N = number of women with a negative HPV test result on the paired clinician-collected sample, among those without CIN2+ after direct referral.

^b^Primary analyses.

**Table 4:** Breakdown of HPV test results by age and cytology, by self-sampling workflow and study population.

	Colposcopy population (CIN2+): positive HPV tests														Primary screening population (<CIN2): negative HPV tests						
	Age (years)						Cytology								Age (years)						
	<30		≥30		P		Negative		Borderline or low-grade abnormal		High-grade abnormal		P		<30		30-49		50-64		P^c^
		% (n/N)^a^		% (n/N)^a^				% (n/N)^a^		% (n/N)^a^	% (n/N)^a^			(n/N)^b^		(n/N)^b^		(n/N)^b^			
Evalyn + cobas		0.962 (25/26)		0.882 (67/76)		0.112		0.857 (6/7)		0.833 (15/18)	0.922 (71/77)	0.263		0.925 (111/120)		0.962 (455/473)		0.979 (230/235)		0.067	
FLOQSwabs+cobas		0.897 (26/29)		0.956 (87/91)		0.117		1 (8/8)		0.889 (8/9)	0.941 (96/102)	0.166		0.895 (137/153)		0.908 (514/566)		0.943 (182/193)		0.423	
Evalyn + APTIMA		0.857 (24/28)		0.724 (42/58)		0.086		0.714 (5/7)		0.850 (34/40)	0.692 (27/39)	0.069		0.979 (94/96)		0.986 (511/518)		0.995 (196/197)		0.003	
FLOQSwabs + APTIMA		0.917 (22/24)		0.929 (39/42)		0.430		0/0		0/0	0.924 (61/66)	-		0.778 (147/189)		0.937 (429/458)		0.902 (111/123)		<0.001	
Multitest + APTIMA		0.931 (27/29)		0.918 (45/49)		0.420		1 (4/4)		0.947 (18/19)	0.909 (50/55)	0.449		0.823 (144/175)		0.871 (507/582)		0.905 (133/147)		0.050	

*CIN* cervical intraepithelial neoplasia, *HPV* human papillomavirus.

^a^Relative sensitivity: n = number of women with a positive HPV test result on the self-collected sample, N = number of women with a positive HPV test result on the clinician-collected referral sample, among those with CIN2+.

^b^Relative specificity: n = number of women with a negative HPV test result on the self-collected sample, N = number of women with a negative HPV test result on the paired clinician-collected sample, among those without CIN2+ after direct referral.

^c^Testing for differences in HPV test result discordance between self- and clinician-collected samples by age group.

### Relative specificity

HPV positivity on the clinician-collected sample in primary screening ranged between 8.2% and 12.3% (Table 2). In the Evalyn+cobas, FLOQSwabs+cobas, and Evalyn + APTIMA workflows, 10.5–14.3% of self-collected samples returned a positive HPV test, which is an increase of ~20% compared with the paired clinician-collected samples. In the FLOQSwabs+APTIMA and Multitest + APTIMA workflows, ~22% of the self-collected samples returned a positive HPV test result, which was around double that of the paired clinician-collected samples. The proportion of inadequate self-collected samples was higher in the FLOQSwabs+cobas workflow: 5.6% compared to the Evalyn+cobas workflow which was 1.0%. The APTIMA workflows had negligible invalid rates, however, this might be because the invalid status in the APTIMA is not influenced by amplification of an endogenous control.

Conditional relative specificity varied by workflow and ranged from 0.87 (90% CI: 0.85–0.89) with the Multitest+APTIMA workflow to 0.99 (90% CI: 0.97-1.01) with the Evalyn+APTIMA workflow (Table 3). When inadequate self-collected samples were excluded from the analysis, the estimate of the relative specificity for the FLOQSwabs + cobas workflow increased from 0.91 (90% CI: 0.90–0.93) to 0.97 (90% CI: 0.96–0.99). After exclusion of women younger than 30 years and those with recent abnormalities, who represented a larger proportion of the recruited women in the FLOQSwabs + APTIMA and Multitest+APTIMA than other workflows, the estimate increased from 0.89 (90% CI: 0.87–0.92) to 0.94 (90% CI: 0.92–0.97) for FLOQSwabs + APTIMA and from 0.87 (90% CI: 0.85–0.89) to 0.89 (90% CI: 0.87–0.91) for Multitest + APTIMA. In general, the concordance between HPV test results on self- vs. clinician-collected samples increased with age (Supplementary Information Table S1).

## Discussion

Four self-sampling workflows including both cobas and APTIMA HPV assays showed ≥84% conditional relative sensitivity for the detection of CIN2+ in a colposcopy referral setting, based on one-sided 95% CIs. One workflow, Evalyn + APTIMA, showed a substantially lower relative sensitivity (≥68%). These estimates appeared robust across a range of subgroups. Estimates of conditional relative specificity varied between ≥85% (Multitest + APTIMA) and ≥97% (Evalyn +APTIMA), also based on one-sided 95% CIs. Relative specificity increased when subgroups with more frequent HPV infections (young women, those with recent abnormalities) and samples that returned an invalid test result were excluded.

The study started recruiting women in 2021 and was completed under challenging circumstances for the NHS and its cancer screening services. The series of lockdown events related to the COVID-19 pandemic led to a backlog of people needing appointments whilst the capacity was reduced, e.g. by precautions related to reduction in risk of COVID-19 transmission. In colposcopy clinics, therefore, fewer women than expected were eligible for the study because the 8-week window since their primary screening test had passed, which was more likely in case they presented with negative or low-grade abnormal cytology. Although these difficulties delayed the completion of the study, the sites recruited to target for the pre-defined plan. In the two workflows that were operated by the London laboratory (FLOQSwabs+APTIMA and Multitest+APTIMA), we observed a higher proportion of women recruited in primary care who had recent abnormalities. This might have been at least partly due to a policy (non-standard setting of the recall date on discharge from colposcopy) that is in use in the London laboratory’s catchment areas; so that women remain on early recall outside of the programme’s guidance and attend for a new clinician-collected sample following an invitation in 12 months. The proportion of women with a positive HPV test on a paired clinician-collected sample ranged between 8% and 12%. These differences were not examined further, but may be related to the women’s age distributions, the HPV vaccination coverage among those attending screening, previous HPV-based screening in the area (e.g. in the English HPV pilot, or during early regional roll-out in 2018-2019 to mitigate cytology capacity), and other factors. The observed differences in women’s characteristics limit the interpretation of direct comparisons between the five self-sampling workflows.

While pre-analytical self-collected sample processing protocols continue to undergo further refinement [10, 31–33], those used within the study were informed by contemporary scientific findings [10, 34]. Nevertheless, the high proportion of samples with invalid HPV test results on one of the workflows underlines the importance of clear laboratory acceptance and rejection criteria which include maximum time from sample collection to receipt in the laboratory. A forthcoming companion manuscript will provide more detail.

Although HPValidate was embedded in the routine setting of the English screening programme, generalisation of the results is not straightforward. Self-collection took place in the clinic environment, with a clinician nearby. Some of the women found this reassuring and reported that advice from a nurse helped them use the device correctly [35]. ‘At-home’ self-collection introduces further variables which can affect the achieved test accuracy and need to be understood prior to a national roll-out of self-sampling, including the adequacy of the instructions for use, and the reliability and timeliness of postal services.

Results of a 2018 meta-analysis, which included predominantly studies undertaken within colposcopy referral settings, indicated that HPV DNA testing using PCR assays might be almost as sensitive for the detection of CIN2+ as on clinician-collected samples (relative detection: 0.99, 95% CI: 0.97-1.02) [36]. The conditional relative sensitivity estimates similarly derived from a referral population in the present study were lower. This was the case even for the workflows relying on the PCR-based cobas DNA assay: 0.90, 90% CI: 0.84–0.94, for Evalyn and 0.94, 90% CI: 0.90–0.97, for FLOQSwabs. One contributing factor is that in HPValidate a concurrent clinician-collected sample was not taken at the time of the colposcopy appointment (for pragmatic reasons and based on clinical steer). Because HPV clearance is common [37], the (up to) 2-month difference in the timing of the two samples could, theoretically, disadvantage the assessment of the self-sampling workflows. However, we expect that if clearance indeed took place between the referral and the colposcopy appointment it would be more likely among women without CIN2+ who were excluded from the analysis of the relative sensitivity [38].

As in most previous studies [36], women with CIN2+ presenting with negative cytology at the time of primary screening were under-represented in most HPValidate workflows due to operational challenges. When they were recruited into the study, they provided their self-collected samples at the colposcopy appointment which took place up to 2 years after the primary screening test. In line with the eligibility for colposcopy referral within the English and other similar screening programmes, these infections were allowed to persist and progress to cytological abnormalities. Persistence may be accompanied with an increase in HPV viral load [39] facilitating detection by the assay [40]. This phenomenon could thus contribute to an optimistic assessment of test sensitivity [27]. Estimates from studies using samples collected at the time of primary screening may result in somewhat lower but more realistic estimates for the intended use of self-collection [7, 8, 27].

The APTIMA mRNA assay is highly sensitive for the detection of high-grade CIN on clinician-collected samples [41], but has occupied a unique position in the discussions regarding HPV self-collection. Prior self-collection evidence appeared to suggest that this assay is less sensitive for the detection of CIN2+ than DNA assays, with a meta-analytic pooled estimate of the relative sensitivity of 0.84 (95% CI: 0.74–0.96) [42]. However, the study with the lowest estimate of the relative sensitivity (0.64, 95% CI: 0.44–0.88) [43] was undertaken in 2009 which is before the threshold for a positive test result was decreased from ≥1.0 s/co to ≥0.5 s/co. The remaining studies all showed relative sensitivity estimates around 0.90 (range: 0.86 [44] to 0.94) [45], but with wide confidence intervals due to their small sizes. In another more recent referral population study from Thailand using the Multitest device, 112 of the 124 women with CIN2+ had a positive clinician-collected test and 108 tested positive with self-collection (relative sensitivity: 0.96) [46]. Nevertheless, the evidence for the use of the APTIMA assay, like that for any HPV DNA assay [7, 8, 31], would be strengthened if its test accuracy were studied in well-screened populations attending primary screening [27].

With respect to clinician-collected samples, APTIMA has shown some evidence of a slightly higher test specificity than HPV DNA assays, with a pooled estimate compared with the Hybrid Capture 2 or GP5+/6+ assays of 1.03 (95% CI: 1.02–1.04) [42]. Self-collected samples showed a similar specificity as clinician-collected samples, with a pooled estimate of the relative specificity of 0.96 (95% CI: 0.91–1.01) [42]. Unlike the two London workflows in our study (using the FLOQSwabs and Multitest devices), which both showed a doubling in test positivity with self-collected vs clinician-collected samples, no previous study reported a notable increase in test positivity with self-collected samples [45–49]. We could not identify an obvious technical issue that could drive the observed discrepancy in HPValidate, but age, screening history and other unmeasured population factors specific to the relevant catchment areas may have played a role. Similar patterns have been suggested in the recent YouScreen study, which offered self-collection to women overdue for screening and was also undertaken in the London catchment area [50]. YouScreen used a FLOQSwabs+cobas self-sampling workflow, and found that only half of the women with a positive self-sampling test went on to have a positive clinician-collected HPV test once they presented for triage; the latter was tested with the APTIMA assay [50]. In this case, some discordance between the self- and clinician-collected samples could be expected because of the different detection targets between the cobas and APTIMA assays [51], and because the two samples were obtained at separate appointments. In HPValidate, the FLOQSwabs+cobas workflow was studied in the area belonging to the Manchester laboratory. Once invalid self-collected samples were excluded from the analysis, the estimated relative specificity of this workflow was high, >0.96. Therefore, to better understand whether the apparently poor specificity of certain workflows is real or an artefact of population characteristics in specific geographies, any future validation should be extended across multiple laboratory areas. Projects should also implement explicit laboratory acceptance criteria to minimise invalidity rate and thus the requirement for repeat samples. All said, performance criteria (both clinical and technical) should arguably be considered with the specific context and application in mind. This may be a more practical and realistic approach than relying on ‘fixed’ threshold targets that self-sampling approaches should achieve irrespective of setting.

In conclusion, test accuracy of the self-sampling workflows included in the HPValidate study seems to vary but in terms of the relative sensitivity, four of them (Evalyn + cobas, FLOQSwabs + cobas or APTIMA, and Multitest + APTIMA) performed relatively close to HPV testing on clinician-collected samples in an English colposcopy referral setting. Further studies in primary screening populations are, however, warranted to better understand the accuracy of self-sampling in the intended-use population.

## Supplementary information

Appendix

## Data Availability

Please direct any reasonable requests for access to the study data to the UK National Screening Committee inbox at UKNSCdhsc.gov.uk.
