# Supplementary material for: HPValidate—human papillomavirus testing with DNA and mRNA assays on self-collected samples in cervical screening: comparison of test characteristics on three self-sampling devices
Source: Br J Cancer. 2025 Jul 8;133(5):665–73. doi: 10.1038/s41416-025-03102-5 (PMC12405571; doi:10.1038/s41416-025-03102-5)
Supplement: Supplementary file 1 — Appendix [file 41416_2025_3102_MOESM1_ESM.docx]

21 September 2024

**SUPPLEMENTARY INFORMATION**

**HPValidate – HUMAN PAPILLOMAVIRUS TESTING WITH DNA AND mRNA ASSAYS ON SELF-COLLECTED SAMPLES IN CERVICAL SCREENING: COMPARISON OF TEST CHARACTERISTICS ON THREE SELF-SAMPLING DEVICES**

Christopher S Mathews,^a^ Alexandra Sargent,^b^ Kate Cuschieri,^c^ Matejka Rebolj,^a^

Adam R Brentnall,^d^ Anne Mackie,^e^ Charlotte Mills,^e^ Carolina Martinelli,^e^ Ann-Marie Wright,^e^ Katherine Hunt,^f^ Andrew Bird,^f^ Hasit Patel,^g^ David Smith,^g^ Trudy Johnson,^h^

Kay Ellis,^h^ Mark Hunt,^i^ Karin Denton^f^

^a^ Centre for Cancer Screening, Prevention, and Early Detection, Wolfson Institute of Population Health, Queen Mary University of London, London, UK

^b^ Cytology Department, Manchester Royal Infirmary, Manchester University NHS Foundation Trust, Manchester, UK

^c^ Scottish HPV Reference Laboratory, Royal Infirmary of Edinburgh, NHS Lothian Scotland, Edinburgh, UK & Centre for Reproductive Health, University of Edinburgh, UK

^d^ Centre for Evaluation and Methods, Wolfson Institute of Population Health, Queen Mary University of London, London, UK

^e^ Primary Care and Prevention, Department of Health and Social Care, London, UK

^f^ Severn Pathology, Southmead Hospital, North Bristol NHS Trust, Bristol, UK

^g^ Health Service Laboratories LLP, London, UK

^h^ NEY Cervical Screening, The NHS Pathology Centre, Queen Elizabeth Hospital, Gateshead Health NHS Foundational Trust, Gateshead, UK

^i^ Cytopathology Department, Norfolk and Norwich University Hospitals NHS Foundation Trust, Norwich, UK

This document includes:

1. Detailed sample size considerations for the study, and
2. Additional tabulation of the study data.

**SAMPLE SIZE CONSIDERATIONS**

For the colposcopy component of the study, the target study size was ≥60 women with CIN2+ per self-sampling workflow, diagnosed at direct colposcopy referral of women with HPV-positive/cytology-abnormal screening samples, or after the 12- or 24-month early recall of women with HPV-positive/cytology-negative screening samples (in these women, primary screening took place before recruitment into the study). Assuming independence between self- and clinician-sampling (i.e., kappa is zero), if both tests have a true absolute sensitivity of 97.5% for CIN2+ detection, then n=60 cases would provide approximately 80% power to reject inferiority of self-sampling using a 90% threshold at the one-sided 5% level. If the absolute sensitivity is 95% for both, then power is approximately 80% when testing non-inferiority at the 85% cut point, and 99% when testing non-inferiority at the 75% cut point. If the absolute sensitivity of clinician-sample HPV testing is 95%, and it is 90% for self-sampling (true relative sensitivity 90/95=94.7%) then n=60 would have approximately 90% power to reject inferiority of self-sampling using a threshold of 75%. Therefore, n=60 cases per self-sampling workflow was felt to provide sufficient information to decide whether to investigate different self-sampling tests in subsequent studies.

Per self-sampling workflow, in order to achieve at least n=60 CIN2+ cases we sought to recruit 350 consecutive consented women in colposcopy clinics based on the findings from the English HPV screening pilot. ^1^ In the pilot, 4.0% of unvaccinated women screened with HPV testing on clinician samples for the first time were referred to colposcopy directly (with a positive predictive value, PPV, for CIN2+ of 43%), 1.0% at the 12-month early recall (PPV: 37%), and 1.4% at the 24-month early recall (PPV: 21%). On average, the PPV of a colposcopy was 37%. For HPValidate, it was conservatively assumed that the PPV could be halved compared to the HPV screening pilot. This is because HPValidate is being undertaken while the screening programme is offering screening to women who were vaccinated against HPV16/18^2^ and because some of the women recruited into the study may have undergone previous HPV tests in areas that introduced HPV-based screening prior to the national roll-out. ^3^ Hence, the study sought to recruit 350 consecutive consented women (including ≥60 CIN2+ cases, resulting in a PPV of ≥17%) attending for colposcopy indicated by routine cervical screening, per self-sampling workflow.

For the primary care component of the study, recruitment of 1000 consecutive consented women per self-sampling workflow was considered achievable. At the time the study was designed, few data from primary screening populations were available. Recently, data were published from a well-screened population in Scotland in which the relative specificity of self-sampling (cobas 4800 testing of samples collected with PCR female swab sample packets) vs. clinician sampling, estimated as the ratio of test-negatives in women without CIN2+, was 0.98 (86% vs. 88%), relative test positivity was 1.17 (14% vs. 12%), and kappa was 0.73. ^4^ A meta-analysis of primary screening studies combining previously well-screened and unscreened populations estimated the test positivity ratio as 1.01 and kappa as 0.65. ^5^ Varying test positivity for self-sampling between 8% (assuming a lower positivity in a partially vaccinated HPValidate population) and 14% and that of clinician sampling between 8% and 12%, and kappa values between ~0.60 and ~0.75, a study size with 1000 recruited women has >90% power to reject inferiority of the relative specificity of self-sampling at the threshold level of 0.95 at the one-sided 5% significance level. The power remains at ≥80% when 800 women are recruited. Only under a handful of scenarios, including lower test positivity values (e.g., 10% vs 8%) combined with higher kappas (e.g., ~0.70) from the ranges specified above, study size of 1000 has >90% power to reject inferiority of individual self-sampling workflows at the threshold of 0.98.

**REFERENCES**

1 Rebolj, M., Mathews, C.S., Pesola, F., Cuschieri, K., Denton, K., Kitchener, H. Age-specific outcomes from the first round of HPV screening in unvaccinated women: Observational study from the English cervical screening pilot. *BJOG* **129**, 1278-1288 (2022).

2 Rebolj, M., Pesola, F., Mathews, C., Mesher, D., Soldan, K. Kitchener, H. The impact of catch-up bivalent human papillomavirus vaccination on cervical screening outcomes: an observational study from the English HPV primary screening pilot. *Br. J. Cancer* **127**, 278-287 (2022).

3 Rebolj, M., Rimmer, J., Denton, K., Tidy, J., Mathews, C., Ellis, K., et. al. Primary cervical screening with high risk human papillomavirus testing: observational study. *BMJ* **364**, l240 (2019).

4 Stanczuk, G.A., Currie, H., Forson, W., Baxter, G., Lawrence, J., Wilson, A., et al. Self-sampling as the principal modality for population based cervical screening: Five-year follow-up of the PaVDaG study. *Int. J. Cancer* **150**, 1350-1356 (2022).

5 Arbyn, M., Castle, P. E., Schiffman, M., Wentzensen, N., Heckman-Stoddard, B., Sahasrabuddhe, V. V. Meta-analysis of agreement/concordance statistics in studies comparing self- vs clinician-collected samples for HPV testing in cervical cancer screening. *Int. J. Cancer* **151**, 308-312 (2022).

Table S1. HPV test results on the paired self- vs. clinician-collected samples in primary care, by age group and self-sampling workflow.

| **Self-sampling workflow and age group (in years)** | **Test result on the SS sample** | **Test result on the paired CS LBC sample** | | | **Concordance** |
| --- | --- | --- | --- | --- | --- |
|  |  | LBC negative | LBC positive | LBC invalid |  |
| **Evalyn+cobas** |  |  |  |  |  |
| **24-29** | SS negative | 109 | 2 | 0 | 90.8% |
|  | SS positive | 10 | 20 | 0 |  |
|  | SS invalid | 1 | 0 | 0 |  |
| **30-49** | SS negative | 448 | 7 | 0 | 93.9% |
|  | SS positive | 20 | 58 | 0 |  |
|  | SS invalid | 5 | 1 | 0 |  |
| **50-64** | SS negative | 228 | 2 | 0 | 96.5% |
|  | SS positive | 5 | 22 | 0 |  |
|  | SS invalid | 2 | 0 | 0 |  |
| **FLOQSwab+cobas** |  |  |  |  |  |
| **24-29** | SS negative | 135 | 2 | 0 | 87.6% |
|  | SS positive | 5 | 13 | 0 |  |
|  | SS invalid | 13 | 1 | 0 |  |
| **30-49** | SS negative | 509 | 5 | 0 | 89.8% |
|  | SS positive | 23 | 34 | 0 |  |
|  | SS invalid | 34 | 0 | 0 |  |
| **50-64** | SS negative | 178 | 4 | 0 | 91.0% |
|  | SS positive | 7 | 13 | 0 |  |
|  | SS invalid | 8 | 0 | 0 |  |
| **Evalyn+APTIMA** |  |  |  |  |  |
| **24-29** | SS negative | 84 | 8 | 2 | 81.2% |
|  | SS positive | 11 | 11 | 0 |  |
|  | SS invalid | 1 | 0 | 0 |  |
| **30-49** | SS negative | 487 | 22 | 2 | 90.5% |
|  | SS positive | 31 | 35 | 0 |  |
|  | SS invalid | 0 | 0 | 0 |  |
| **50-64** | SS negative | 188 | 7 | 1 | 92.1% |
|  | SS positive | 9 | 10 | 0 |  |
|  | SS invalid | 0 | 0 | 0 |  |
| **FLOQSwab+APTIMA** |  |  |  |  |  |
| **24-29** | SS negative | 139 | 8 | 0 | 75.8% |
|  | SS positive | 50 | 43 | 0 |  |
|  | SS invalid | 0 | 0 | 0 |  |
| **30-49** | SS negative | 415 | 14 | 0 | 88.8% |
|  | SS positive | 43 | 36 | 0 |  |
|  | SS invalid | 0 | 0 | 0 |  |
| **50-64** | SS negative | 111 | 0 | 0 | 90.8% |
|  | SS positive | 12 | 7 | 0 |  |
|  | SS invalid | 0 | 0 | 0 |  |
| **Multitest+APTIMA** |  |  |  |  |  |
| **24-29** | SS negative | 142 | 2 | 0 | 84.2% |
|  | SS positive | 33 | 45 | 0 |  |
|  | SS invalid | 0 | 0 | 0 |  |
| **30-49** | SS negative | 496 | 10 | 1 | 84.5% |
|  | SS positive | 86 | 34 | 0 |  |
|  | SS invalid | 0 | 0 | 0 |  |
| **50-64** | SS negative | 133 | 0 | 0 | 91.1% |
|  | SS positive | 13 | 10 | 0 |  |
|  | SS invalid | 1 | 0 | 0 |  |

Abbreviations. CS: clinician-collected sample. LBC: liquid-based cytology sample. SS: self-collected sample.
